# A Predictive Model for Steady State Ozone Concentration at an Urban-Coastal Site

**DOI:** 10.3390/ijerph16020258

**Published:** 2019-01-17

**Authors:** Mansour A. Alghamdi, Afnan Al-Hunaiti, Sharif Arar, Mamdouh Khoder, Ahmad S. Abdelmaksoud, Hisham Al-Jeelani, Heikki Lihavainen, Antti Hyvärinen, Ibrahim I. Shabbaj, Fahd M. Almehmadi, Martha A. Zaidan, Tareq Hussein, Lubna Dada

**Affiliations:** 1 Department of Environmental Sciences, Faculty of Meteorology, Environment and Arid Land Agriculture, King Abdulaziz University, P.O. Box 80208, Jeddah 21589, Saudi Arabia; mghamdi2@kau.edu.sa (M.A.A.); khoder_55@yahoo.com (M.K.); asabdelmaksoud@yahoo.com (A.S.A.); hjeelani@gmail.com (H.A.-J.); ishabbaj@yahoo.com (I.I.S.); fmehmadi@gmail.com (F.M.A.); 2 Department of Chemistry, University of Jordan, Amman 11942, Jordan; a.alhunaiti@ju.edu.jo (A.A.-H.); s.arar@ju.edu.jo (S.A.); 3 Finnish Meteorological Institute, Erik Palménin aukio 1, FI-00101 Helsinki, Finland; heikki.lihavainen@fmi.fi (H.L.); antti.hyvarinen@fmi.fi (A.H.); 4 Institute for Atmospheric and Earth System Research (INAR), University of Helsinki, FI-00014 Helsinki, Finland; martha.zaidan@helsinki.fi (M.A.Z.); tareq.hussein@helsinki.fi (T.H.); 5 Department of Physics, University of Jordan, Amman 11942, Jordan

**Keywords:** chemical coupling, nitrogen oxides, ozone, weekend effect

## Abstract

Ground level ozone (*O*_3_) plays an important role in controlling the oxidation budget in the boundary layer and thus affects the environment and causes severe health disorders. Ozone gas, being one of the well-known greenhouse gases, although present in small quantities, contributes to global warming. In this study, we present a predictive model for the steady-state ozone concentrations during daytime (13:00–17:00) and nighttime (01:00–05:00) at an urban coastal site. The model is based on a modified approach of the null cycle of *O*_3_ and *NO_x_* and was evaluated against a one-year data-base of *O*_3_ and nitrogen oxides (*NO* and *NO*_2_) measured at an urban coastal site in Jeddah, on the west coast of Saudi Arabia. The model for daytime concentrations was found to be linearly dependent on the concentration ratio of *NO*_2_ to *NO* whereas that for the nighttime period was suggested to be inversely proportional to *NO*_2_ concentrations. Knowing that reactions involved in tropospheric *O*_3_ formation are very complex, this proposed model provides reasonable predictions for the daytime and nighttime concentrations. Since the current description of the model is solely based on the null cycle of *O*_3_ and *NO_x_*, other precursors could be considered in future development of this model. This study will serve as basis for future studies that might introduce informing strategies to control ground level *O*_3_ concentrations, as well as its precursors’ emissions.

## 1. Introduction

Tropospheric ozone (*O*_3_) is known for causing severe health effects and having environmental impacts [1,2]. Among other photochemical oxidants, *O*_3_ is one of the widely studied subjects worldwide under the category of air pollution. Besides that, *O*_3_ is a key precursor of hydroxyl radicals (*OH*), which control the oxidizing power of the lower atmosphere and by that alters its chemical properties [3].

**Figure 1:**
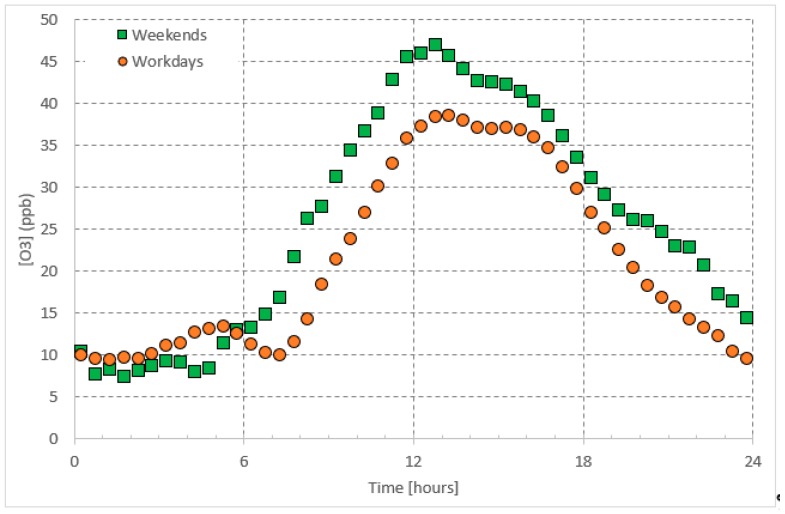
Average daily pattern of *O*_3_ presented separately for workdays and weekends.

Ground level *O*_3_ formation depends on photochemistry, meteorological conditions, and air mass transport [4,5,6,7]. For instance, *O*_3_ is found to peak during the summer time accompanying high temperatures and long daytime hours and thus seems to be correlated with solar radiation intensity [8,9,10,11,12,13]. In urban environments, the diurnal cycle of *O*_3_ consists of nighttime low concentrations and daytime high concentrations, which may last for several hours (Figure 1). This high *O*_3_ concentration during the daytime is mainly attributed to photochemical reactions mainly within the *NO_x_–O*_3_ cycle. The low *O*_3_ concentrations during nighttime are the result of the pause in ozone production, due to the absence of photochemical reactions. Eventually, the *O*_3_ is recycled through chemical reactions or is lost by deposition [14]. It is interesting that the daytime steady-state *O*_3_ concentration on weekends is higher than that on workdays. The latter can be attributed to higher traffic on workdays than on weekends, releasing more *NO_x_*, which in turn uses up the daytime available ozone, leaving behind a lower concertation of steady state ozone on workdays. The aforementioned assumptions are discussed in more detail in the following sections. Being the major source of daytime ground level *O*_3_, we believe that the *NO_x_*–*O*_3_ null cycle, can be applied to predict the steady-state daytime *O*_3_ concentrations in urban areas.

The momentary change rate of *O*_3_ concentrations can be described by its sources and sinks involved in atmosphere [15,16]. For instance, in urban environments, *O*_3_ is formed through a series of daytime reactions that involve *NO_x_* (*NO* and *NO*_2_), which are of anthropogenic origin. Other sources of *O*_3_ include volatile organic compounds (*VOCs*) and carbon monoxide (*CO*) [17]. The priority of the reactions depends on the concentrations of *NO_x_* and *VOCs*, as well as the ratio of the two (*NO_x_*/*VOC*) [18]. Accordingly, two regimes for *O*_3_ formation have been proposed. The first one is the *NO_x_*-sensitive regime in which the increase in *NO_x_* concentration causes an increase in *O*_3_ concentration and the formation of *O*_3_ is mainly independent of the *VOCs* concentration. The second one is the *VOC*-sensitive regime in which the *O*_3_ formation is solely dependent on the *VOCs* concentration [19,20]. Therefore, the prevailing regime is specific to the dominant environmental conditions.

**Figure 2:**
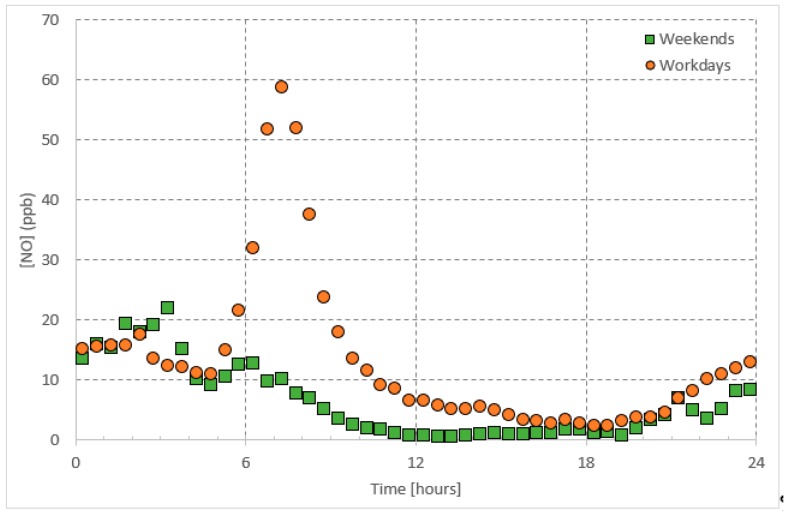
Average daily pattern of *NO* presented separately for workdays and weekends.

**Figure 3:**
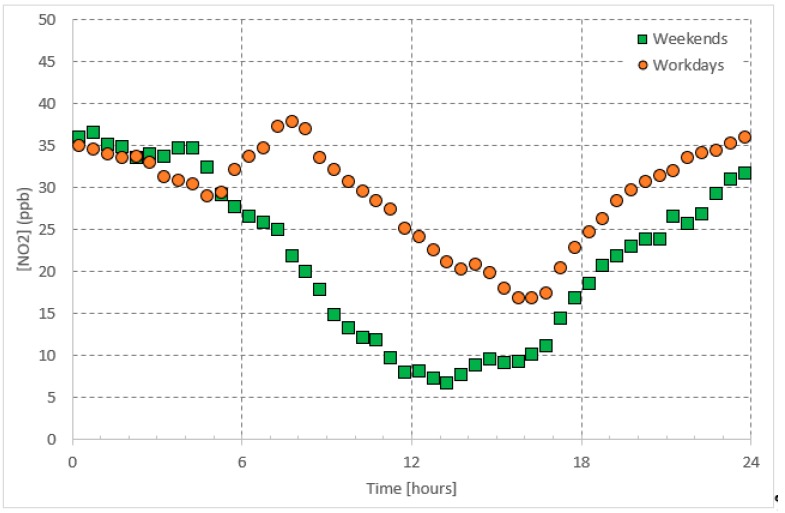
Average daily pattern of *NO*_2_ presented separately for workdays and weekends.

In the urban atmosphere, *NO* and *NO*_2_ are emitted from anthropogenic activities, including combustion processes (e.g., traffic and industrial activities). Their daily patterns (Figure 2 and Figure 3) are, therefore, controlled by these emissions [21,22,23]. Since *NO* is a primary pollutant and acts to form *NO*_2_ upon a series of reactions [24], the *NO*_2_ morning peak appears one hour later than the *NO* peak. The *NO_x_* concentrations vary between morning and evening and the change is attributed to many factors. First, during the early hours of daytime, high traffic emissions are accumulated in the atmosphere when the photo-chemically produced *O*_3_ concentrations are still low; *O*_3_ acts as a sink for both *NO* and *NO*_2_. Concurrent with sunrise, these pollutants are consumed with daytime produced *O*_3_ and are subject to thermal turbulence, due to higher temperature resulting in their dilution, dispersion within expansion in the boundary layer and eventually a drop in their concentrations [25,26]. On the other hand, along with sunset *NO* and *NO*_2_ encounter lower temperature, less boundary layer mixing and low dispersion leading to an increase in their concentrations.

The characteristics and patterns of ground level *O*_3_ have been the subject of many studies worldwide [27]. Specifically, the chemical coupling between *O*_3_ and its precursors (*NO* and *NO*_2_) was investigated thoroughly in urban environments [19,22,28,29,30,31]. However, very few studies considered modelling of ground level *O*_3_ [32,33,34,35]. In fact, *O*_3_ is involved in many chemical reactions that sometimes make its prediction very difficult. In this study, we present a simple statistical predictive model to calculate the steady-state daytime and nighttime *O*_3_ concentrations at an urban coastal site. For the purpose of model evaluation, we utilized a one-year data-base of ozone (*O*_3_) and nitrogen oxides (*NO* and *NO*_2_) measured in Jeddah, which is located on the western part of Saudi Arabia [36]. Our model could be modified to evaluate ozone in other urban environments with similar diurnal patterns. 

## 2. Materials and Methods

### 2.1. Simple Statistical Predictive Model

In the troposphere, ozone (*O*_3_) and nitrogen oxides (*NO_x_*) undergo a well-known null cycle in which each gaseous species maintains a steady-state concentration [37]; i.e., balanced production and loss rates balance each other (Figure 1, Figure 2 and Figure 3). As postulated in the introduction, the daytime steady-state *O*_3_ concentration is higher than that during the nighttime steady-state concentrations. Furthermore, the chemical reactions involved with the *O*_3_ are different during both periods. Therefore, we postulate the simple predictive model for two time periods: Daytime and nighttime.

### 2.2. Daytime Steady-State O_3_ Concentrations Prediction

Under atmospheric conditions and in the presence of solar radiation (*λ* < 424 nm), the *O*_3_–*NO_x_* null cycle includes three successive reactions [37]:

(1) $$NO_{2}+hv\to NO+O,\;O+O_{2}+M\to O_{3}+M^{*},\;O_{3}+NO\to NO_{2}+O_{2},$$

where *M* is an inert ground state (either *N*_2_ or *O*_2_) that acts as a surface for the reaction to take place and M* is the excited state of the molecule, *hv* is the energy of the solar radiation photons that induces photochemical oxidation, *O* is known to be highly reactive and disappears as soon as it is generated. Here, the concentration of *O*_2_ is assumed to be constant.

Under steady-state conditions, the null cycle has the steady-state formula,

(2) $$\frac{J_{NO_{2}}}{k_{3}}=\frac{\left[NO\right]\left[O_{3}\right]}{\left[NO_{2}\right]}\;,$$

where *J_NO2_* is the rate coefficient of *NO*_2_ photolysis, *k*_3_ is the reaction rate coefficient of *O*_3_ and *NO*. It is well known that the *k*_3_ is temperature dependent [38]; *k_3_ =* 3.23 exp(−1430/T) in units of ppb^−1^min^−1^. However, the seasonal temperature variation is few degrees; and therefore, we do not expect *k*_3_ to have a considerable variation throughout the year in Jeddah.

Re-arrangement of Equation (2) yields a simple equation to predict the concentration of *O*_3_ from the ratio of *NO*_2_ to *NO* concentrations during daytime,

(3) $$\left[O_{3}\right]=\;\alpha \frac{\left[NO_{2}\right]}{\left[NO\right]}+\;\delta_{1},$$

where αis a constant equivalent to *J*_NO2_/*k*_3_ and *δ*_1_(ppb)is also constant related to the background *O*_3_ concentrations (e.g., migrates from the stratosphere to the troposphere, long-range transport, product of other reactions).

During daytime steady-state, using Equation (1):

(4) $$\frac{d\left[NO_{2}\right]}{dt}=-J_{NO_{2}}\left[NO_{2}\right]+k_{3}\left[O_{3}\right]\left[NO\right]=0,$$

Upon rearranging we get Equation (2). We then compute a linear regression of [*O*_3_] vs. [*NO*_2_/*NO*] of measured data. We, thus, are able to derive the constants for the model as *y* = a*x* + b (Equation (3)), where a is a constant equivalent to *J_NO2_*/*k*_3_ and b is also constant related to the background *O*_3_ concentrations.

### 2.3. Nighttime Steady-State O_3_ Concentrations Prediction

During night-time hours, *O*_3_ is mainly consumed through its reaction with *NO*_2_,

(5) $$O_{3}+NO_{2}\to NO_{3}+O_{2},$$

Applying reaction rate kinetics and rearrangement of the Equation (4) yields a simple equation to predict the nighttime *O*_3_ based on the concentration of its major nighttime sink compound *NO*_2_,

(6) $$\left[O_{3}\right]=\beta \frac{1}{\left[NO_{2}\right]}-\;\delta_{2},$$

where *β*(ppb^2^) is a constant equivalent to the reaction rate of *O*_3_ with *NO*_2_ and *δ*_2_(ppb) is again a constant related to the background *O*_3_ concentrations during the night.

During nighttime, Equation (4) steady state conditions are:

(7) $$\frac{d\left[O_{3}\right]}{dt}=k_{\left(NO_{2},O_{3}\right)}\left[O_{3}\right]\left[NO_{2}\right]=0,$$

Upon rearranging we get Equations (6) and (7). We then compute a linear regression of [*O*_3_] vs. [*NO*_2_] of measured data. We, thus, are able to derive the constants for the model as *y* = a*x* + b (Equation (3)), where a is a constant equivalent to *k* (reaction rate of *O*_3_ with *NO*_2_) and *b* is again a constant related to the background *O*_3_ concentrations.

### 2.4. Data-Base

In this study, we utilized a one-year data-base of *O*_3_ and *NO_x_* concentrations measured at an urban site in Jeddah, Saudi Arabia between 1 January and 31 December 2012 [36]. The data-base is utilized to only evaluate the above described simple predictive model for steady-state *O*_3_ concentrations. The measurement was conducted at the King Abdul-Aziz University (KAU) campus, which is surrounded by major roads and a highway. Jeddah itself is situated on the west coast of Saudi Arabia and is considered the largest sea port on the Red Sea. Potential sources of air pollution in the city are mainly vehicle emissions (1.4 million vehicles; [39]) and industrial (oil refinery, desalination plant, power generation plant, and manufacturing industry). A lot of these emissions act as *O*_3_ precursors; under favored meteorological conditions and abundance of solar radiation, which are available in Jeddah.

## 3. Results

### 3.1. Overview of the Daily Patterns

The *O*_3_ concentrations showed a clear daily pattern with high concentrations during the daytime, which was as high as 39 ppb and 47 ppb on workdays (Saturday–Wednesday) and weekends (Friday), respectively (Figure 1). The nighttime (before 05:00) concentrations were between 7.5 ppb and 13.2 ppb. As mentioned before in the introduction section, higher *O*_3_ concentrations on weekends daytime are not only attributed to the *NO_x_* cycle, but also possibly due to differences in the concentrations of other precursors (e.g., *CO* and *VOC)*. The presence of *VOCs* changes the path of *O*_3_ formation by altering the *NO_x_* cycle mechanism through reactions of hydroxyl radicals, which in turn oxidize *NO* without the use of *O*_3_. The latter, along with the photolysis of *NO*_2_, leads to accumulation of *O*_3_ during the daytime on weekends. Furthermore, when *NO_x_* concentrations are high, the reaction of *NO*_2_ and *OH* to give *HNO*_3_ is favored [17], which reduces the *NO*_2_ concentrations available for photolysis. In turn, this leads to low photolysis rate *J_NO2_* during the weekends.

**Figure 4:**
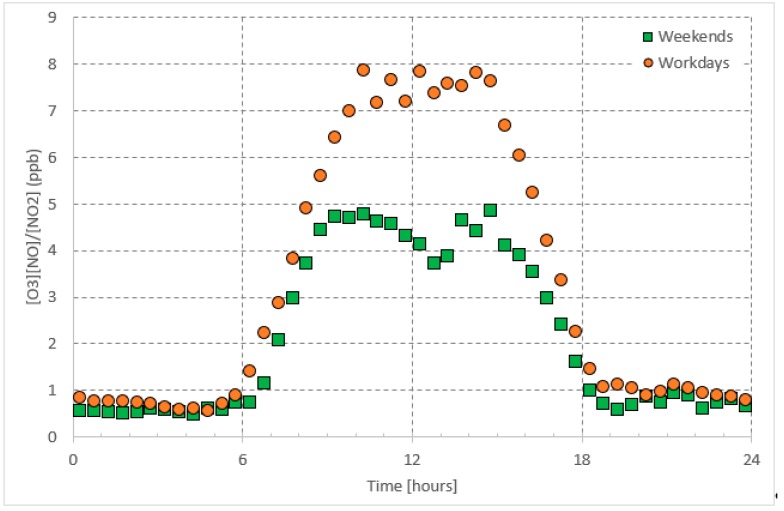
Average daily pattern of photo-stationary state concentrations [*NO*][*O*_3_]/[*NO*_2_] presented separately for workdays and weekends.

**Figure 5:**
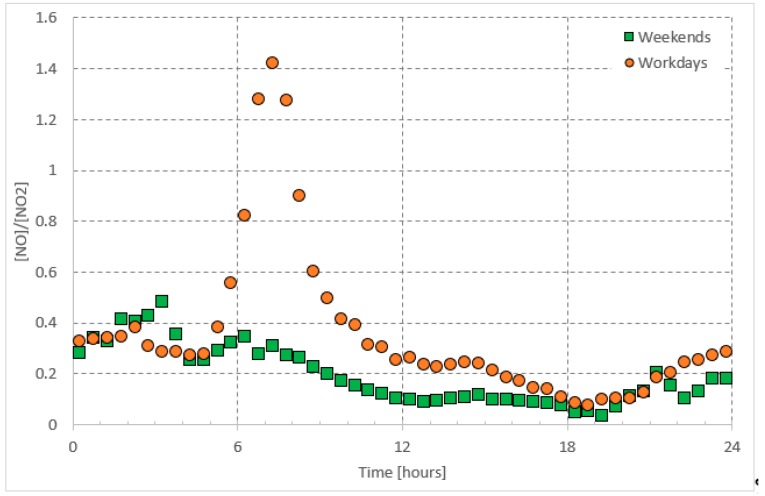
Average daily pattern of [*NO*]/[*NO*_2_] presented separately for workdays and weekends.

Recalling Equation (2), the daily pattern of *J_NO2_/k*_3_ (represented by the concentrations ratio [*O*_3_][*NO*]/[*NO*_2_]) is characterized by a double peak (before noon and in the afternoon). The nighttime value varied between 0.5 and 1 ppb. The daytime value was as high as 5 ppb on weekends and as high as 8 on workdays (Figure 4). As claimed before, *k*_3_ does not have significant differences throughout the year in Jeddah; and thus, the daily pattern, shown in Figure 4, should represent the daily pattern of *J_NO2_*. In general, *J_NO2_* is the rate of photolysis of *NO*_2_ and it seems to be lower on weekends than on workdays. In general, it has been well known that photolysis occurs more rapidly during lower PM (particulate matter) concentrations; This is mainly observed during the weekends [31,40]. The reason could be also referred to the change of both the [*NO*]/[*NO*_2_] ratio and the *O*_3_ concentration (‘weekend effect’). As shown in Figure 5, the daytime value of [*NO*]/[*NO*_2_] is higher on workdays than on weekends.

### 3.2. Prediction of Steady-State O_3_ Concentration

As shown in Figure 1, Figure 2 and Figure 3, regarding the daily pattern of *O*_3_ and *NO_x_*, the steady-state conditions are met during 13:00–17:00 (referred to as daytime steady-state period) and 01:00–05:00 (referred to as nighttime steady-state period). We considered the 30-minutes average of the data-base and selected these time periods separately to apply the simple predictive model, which is a linear regression model. We applied the fitting to the whole data set. The nighttime period for all weekdays was considered as one period whereas the daytime period was considered separately for workdays (Saturday–Wednesday) and weekends (Friday).

The *O*_3_ concentration prediction for the daytime period according to Equation (3) is best represented by:

(8) $${\left[O_{3}\right]}_{\mathrm{daytime}}=\{\begin{array}{cc} 1.09\frac{\left[NO_{2}\right]}{NO}+29.35 & Workdays\;\left(R^{2}=0.37\right)\;\\ 0.50\frac{\left[NO_{2}\right]}{NO}+35.47 & Weekends\;\left(R^{2}=0.31\right) \end{array}$$

**Figure 6:**
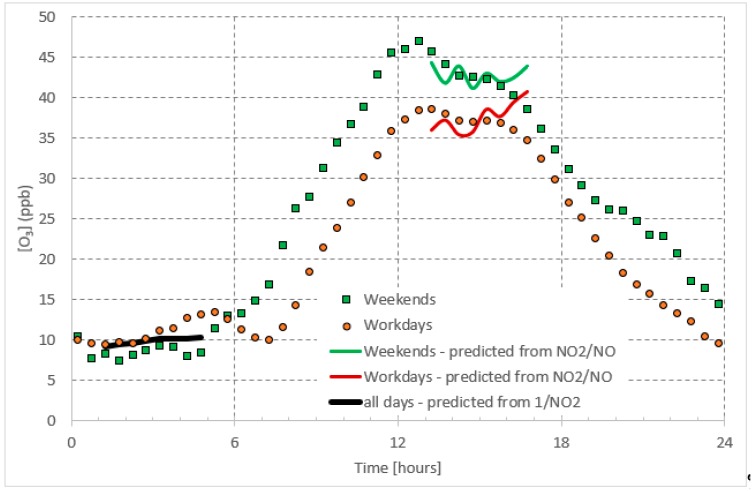
Prediction of daytime and night time *O*_3_ concentrations compared with the measured ones.

The predicted *O*_3_ concentrations based on these equations are shown and compared to the measured ones in Figure 6. Note that the regression model parameters were obtained based on the 30-minutes average of the *O*_3_ and *NO_x_* data-base. In addition, the model predictions were also based on the 30-minutes average of the concentrations and Figure 6 is based on averaging the results to obtain the daily patterns.

Based on Equation (6), the *α* constant, which is supposed to be equivalent to *J*_NO2_/*k*_3_, is found to be 1.09 ppb and 0.50 ppb for workdays and weekends daytime, respectively. The *δ* constant, which is related to the background ozone concentrations, is 29.35 ppb and 35.47 ppb for workdays and weekends, respectively. The theoretical value of *J*_NO2_/*k_3_* calculated from the kinetics of the daytime reactions involved in *O*_3_ formation at steady-state are presented by the function is found to be 8.7 ppb. This can be easily verified for *J*_NO2_ provided by ACOM online database (http://cprm.acom.ucar.edu/Models/TUV/Interactive_TUV/) and substituting *k*_3_ as proposed with Equation (2).

This means that *α* value is different than the ideal one represented by *J*_NO2_/*k*_3_. Note that the kinetic model represents the ideal case, when the concentration of *O*_3_ depends solely on the *NO_x_*–*O*_3_ cycle with no contribution from additional sources or the involvement of other precursors in the *O*_3_ formation processes. Additionally, the ideal case occurs in full solar exposure, without factors leading to solar radiation attenuation, including daytime PM and cloudiness. Also note that the additional parameter *δ*_1_ can be thought of as a parameter that accounts for other processes contributing to the *O*_3_ formation in Jeddah. Interestingly, the value of *δ*_1_ is higher on weekends than on workdays. Other parameters which contribute to δ_1_ include long range transport of *O*_3_, as well as stratosphere-troposphere *O*_3_ migration. The latter is aided by the high temperature in Jeddah which enables this irreversible phenomenon to occur by increasing boundary layer height favoring proper mixing [41].

The *O*_3_ concentration prediction for the nighttime period according to Equation (5) is best represented by,

(9) $${\left[O_{3}\right]}_{nighttime}=\frac{267.01}{\left[NO_{2}\right]}+1.16\;\;\;\;\;\;\;\;\;\;\;\;\;\;\;\;\;\;\;\;\;\;All\;days\left(R^{2}=0.58\right)$$

The predicted *O*_3_ concentrations are also shown and compared to the measured ones in Figure 6. Again, the regression model parameters were obtained based on the 30-minutes average of the *O*_3_ and *NO_x_* data-base.

This equation is based on the fact that *NO*_2_ acts as a major sink for the night-time *O*_3_ [24]. Here the parameter *β* can be thought of as the reaction rate of *O*_3_ with *NO*_2_. In our analysis, *β* is rather similar for all days of the week and its value is about 267 ppb^2^. The second parameter *δ*_2_ has a value of 1.16 ppb. The theoretical value for the reaction rate of *O*_3_ with *NO*_2_ during nighttime is about 1250 ppb^2^ [42,43,44]. Again, the deviations between *β* and the reaction rate of *O*_3_ with *NO*_2_ during nighttime can be explained by the occurrence of additional sinks of ozone, including surface reactions of particulate matter and deposition [24].

## 4. Conclusions

In this study, we suggested a simple statistical predictive model to calculate the steady-state daytime and nighttime *O*_3_ concentrations at an urban coastal site. This model was formulated based on a modified approach of the null cycle of *O*_3_ and *NO_x_*. The model evaluation was performed by utilizing a one-year data-base of ozone (*O*_3_) and nitrogen oxides (*NO* and *NO*_2_) measured in Jeddah, which is located on the west coast of Saudi Arabia. The steady-state conditions for *O*_3_ and *NO_x_* at this site were observed during daytime (13:00–17:00) and nighttime (01:00–05:00).

The simple model for daytime concentrations was proposed to be linearly dependent on the concentration ratio of *NO*_2_ to *NO* whereas that for the nighttime period it was suggested to be inversely proportional to *NO*_2_ concentrations. Since the daytime *O*_3_ concentrations on workdays (Saturday–Wednesday) were lower than those on weekends (Friday), two separate formulas were suggested for the daytime concentration predictions. Recalling the complex reactions involved in tropospheric *O*_3_ formation, this proposed simple model provided reasonable predictions for the daytime and nighttime concentrations. Since the current description of the model is solely based on null cycle of *O*_3_ and *NO_x_*, other precursors should be considered in future development of this simple model.

Our study could be applied to several urban environments with similar emission patterns, as well as fill the gaps in *O*_3_ data when no measurements were collected. Our study could also serve as basis for future studies for enforcing strategies to control ground level *O*_3_ concentrations, as well as its precursors’ emissions in polluted environments.
